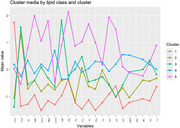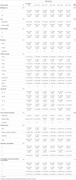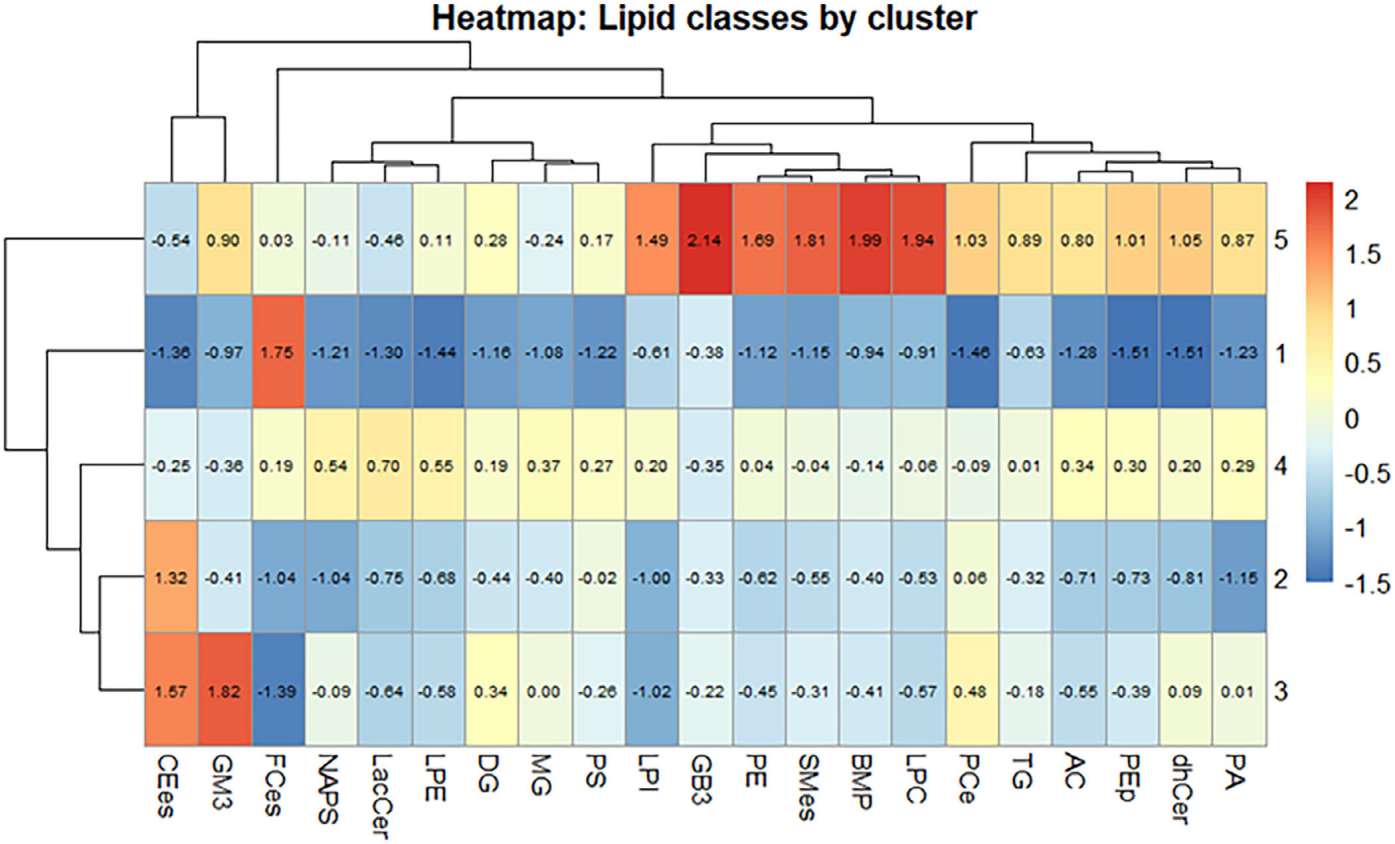# Differential lipid profile for predicting Alzheimer's disease risk in a cohort of patients with PSEN1‐E280A mutation

**DOI:** 10.1002/alz70856_105806

**Published:** 2026-01-08

**Authors:** Nelson D. Galvis‐Garrido, Juan Pablo Pablo Barbosa‐Carvajal, Laura Alejandra Lozano‐Trujillo, Ivan Daniel Salomón‐Cruz, Kenneth S. Kosik, Daniel C. Aguirre‐Acevedo, David Fernando Aguillón Niño, Estela Area‐Gomez, Gloria Patricia Cardona Gomez

**Affiliations:** ^1^ Grupo de Neurociencias de Antioquia, Medellín, Antioquia, Colombia; ^2^ University of California Santa Barbara, Santa Barbara, CA, USA; ^3^ Grupo de Neurociencias de Antioquia, Facultad de Medicina, Universidad de Antioquia, Medellín, Antioquia, Colombia; ^4^ Columbia University Irving Medical Center, New York City, NY, USA

## Abstract

**Background:**

Alzheimer's disease (AD) is a multifactorial condition influenced by modifiable and non‐modifiable risk factors. It affects approximately 50 million people globally, a number that is expected to triple by 2050 due to aging populations. Identifying early biological changes associated with AD is essential to designing primary interventions. In this context, fluid biomarkers hold significant potential, as they can offer insights into the disease's earliest stages. This study proposes serum lipid signatures as biomarkers for AD risk in preclinical stages.

**Method:**

The study included a cohort of 320 participants clinically and genetically characterized. Genetic profiling included APOE isoforms and the PSEN1‐E280A variant, which is pathogenic for early‐onset familial AD. To classify population subgroups, a Latent Profile Analysis (LPA) model was applied using the “mclust” package in RStudio. This approach used Bayesian statistical models and multiple variables to create distinct profiles based on lipidomic data obtained via high‐resolution mass spectrometry (HR‐Mass Spect), remaining blind to clinical information. Additionally, 192 samples were analyzed for protein biomarkers NFL, GFAP, pTau 231, and pTau 217 using the SIMOA ultrasensitivity technology.

**Result:**

The analysis identified five distinct lipid profiles. Of these, two profiles were associated with an increased genetic risk of AD, while one profile suggested protective effects. Similar results were observed in the analysis performed exclusively on children in an age and sex‐dependent mode.

On the other hand, protein biomarkers were unable to distinguish genetically at‐risk individuals under the age of 20. Furthermore, the lipid analysis provided valuable insights into lipid classes that may be linked to both aging and neurodegenerative processes, further supporting their utility as early biomarkers.

**Conclusion:**

These findings underscore the potential of lipidome as a powerful tool for early detection of AD risk, offering significant advantages over traditional protein biomarkers. Lipid profiles were able to discriminate between genetic risk groups decades before protein biomarkers showed similar sensitivity, highlighting their value in preclinical identification. Additionally, the study suggests that specific lipid pathways may serve as enzymatic targets for designing primary prevention strategies, offering new avenues for intervention in populations at risk for Alzheimer's disease.